# Accidental Organophosphate Poisoning in a Toddler: A Case Report

**DOI:** 10.7759/cureus.63027

**Published:** 2024-06-24

**Authors:** Riya Patel, Sanket Patel, Aakash Verma, Himanshi Baid

**Affiliations:** 1 Pediatrics, Nootan Medical College and Research Centre, Gujarat, IND; 2 Emergency Medicine, Gujarat Adani Institute of Medical Sciences, Bhuj, IND; 3 Emergency Medicine, Command Hospital Air Force, Bangalore, Bangalore, IND; 4 Emergency Medicine, Himalayan Institute of Medical Sciences, Dehradun, IND

**Keywords:** accidental poisoning, pediatric population, organophosphate poisoning, toxicology, emergency medicine

## Abstract

While accidental poisoning is fairly common in children, the data are sparse when organophosphate (OP) is considered the culprit toxin. Only case reports of such patients from the Southeast Asian Region have been documented, despite it contributing largely to the global burden of organophosphorus poisoning in the adult population. This can be attributed to difficulty in diagnosing children because of varied presentations in the pediatric population and unreliable or unavailable exposure history. We present a case of a 19-month-old toddler who presented to the ED with OP poisoning, which proved to be a diagnostic and management challenge because of more common differentials and the unavailability of a clear history.

## Introduction

Organophosphates (OPs) are widely used insecticides worldwide and are one of the leading causes of death because of poisoning in developing nations, especially in South Asia [1]. An estimated 150,000 deaths worldwide are attributed to pesticide poisoning each year, according to the World Health Organization. India accounts for 70,000 of these deaths annually or around 47% of all deaths globally [1,2].

Since pharmacological intervention is required to break the spontaneous reversible link between the OP and cholinesterase, these chemicals function as irreversible cholinesterase inhibitors. Cholinergic toxidrome is the result of excessive activation of both muscarinic and nicotinic acetylcholine receptors, which is brought upon by the buildup of acetylcholine and an increase in synaptic acetylcholine levels caused by this inhibition [3,4].

Although accidental poisoning is fairly common in children, data are variable as per geographical locations when OP is considered as the culprit toxin. The US has reported only 550 cases in patients less than 12 years in 2020 [5]. Only a few cases of such patients from the Southeast Asian region have been reported [6,7]. This can be attributed to difficulty in diagnosing children because of varied presentations in the pediatric population and unreliable or unavailable exposure history. Hence, we would like to present a case of accidental OP poisoning in a 19-month-old toddler who presented to the ED of a tertiary care hospital in India.

## Case presentation

A 19-month-old previously healthy male child was brought into the ED with complaints of cough and cold for two days and difficulty in breathing for the preceding 12 hours.

The primary assessment revealed a rectal temperature of 100.3 F, a heart rate of 190/min, respiratory rate of 40/min, and oxygen saturation (SpO_2_) of 85% on room air with severe respiratory distress and labored breathing. He had pinpoint pupils and profuse bronchial secretions. His Glasgow Coma Scale (GCS) score was nine on arrival at the ED. Respiratory examination revealed bilateral conducted sounds. Emergency airway management was done. This comprised immediate oropharyngeal suction, endotracheal intubation, and assisted ventilation. The patient was also started on IV fluids and antibiotics.

Routine investigations were sent urgently, including blood gas analysis, chest X-ray, a complete hemogram, renal function test, and liver function test.

Because of excessive bronchial secretions and pinpoint pupils, serum cholinesterase level was sent, which was reduced to 1,842 u/L (normal: 3,930-11,500 u/L). A working diagnosis of OP poisoning was established. Further and directed questioning from the parents revealed suspected exposure to an OP compound insecticide from toys (the toys were kept out in the open in the fields where pesticides were sprayed regularly). Dermal decontamination of the patient was done immediately (by removal of all the clothes and wet sponging of the patient).

Gastric lavage revealed clear fluid. Laboratory investigation shows polymorphonuclear leukocytosis (WBC 12,800/cu mm; normal: 4,000-10,000/cu mm) and normal renal and liver function tests. Arterial Blood gas analysis showed a pH of 7.3, partial pressure of carbon dioxide (pCO_2_) of 32.1 mmHg, partial pressure of oxygen (pO2) of 161.8, and bicarbonate (HCO_3_) of 16.7 mmol/L. The coagulation profile was slightly deranged with an international normalized ratio (INR) of 1.8. The blood glucose level was 133 mg/dL (70-110 mg/dL). The chest X-ray, 2D echo, and CT brain showed no abnormalities.

**Figure 1:**
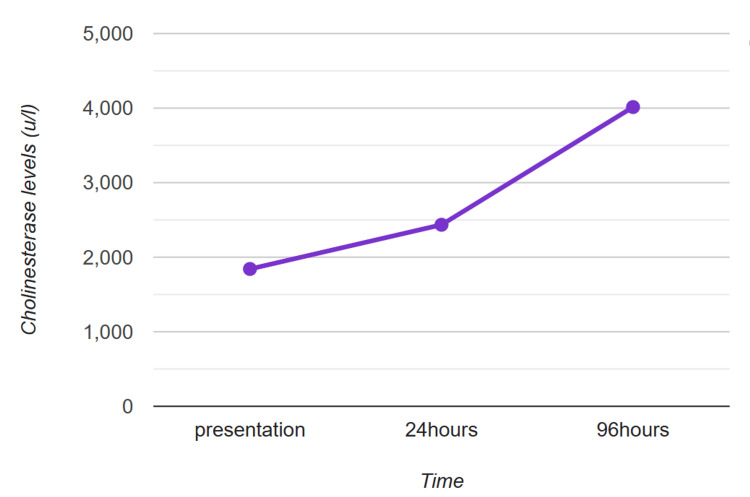
A line graph demonstrating pseudocholinesterase levels over time

The patient was first given an injection of atropine 0.05 mg/kg bolus; however, because of increasing tachycardia, three further doses were given 20 minutes apart. A loading dose of injection pralidoxime (PAM) was given at a dose of 25 mg/kg over one hour and was repeated at 12-hour intervals with two more doses. Serum cholinesterase level was repeated after 24 hours of loading dose showed improvement (Figure 1).

The patient gradually improved with supportive management in the ICU over the next six days. He was weaned off ventilatory support on the sixth day of admission. However, during the ICU stay, the patient developed ventilator-associated pneumonia for which IV antibiotics were initiated and continued.

He was discharged after 19 days of hospital stay with no residual sequelae (both pulmonary and neurological). The patient was followed up after a month, and during that visit, he showed no delayed signs of neurological deficits.

## Discussion

An exposure history and the telltale symptoms of a cholinergic overdose are the primary factors used to diagnose OP poisoning. However, a history of exposure may not be evident sometimes, especially in pediatric cases as we saw in our case [6]. It was only after directed history-taking that, a possible source of insecticide exposure was identified. The severity of intoxication can be related to the type of receptor activity that dominates the clinical presentation. At lower doses of OPs, muscarinic symptoms usually predominate. At higher doses, nicotinic and central muscarinic activity may become prominent. Thus, tachycardia and hypertension can be important signs of severe poisoning as was seen in our case and the presence of these features should not delay therapy or confuse the clinician who traditionally expects the patient to have bradycardia [7,8]. The characteristic complex of symptoms (lacrimation, salivation, urination, and diarrhea) is an unreliable indicator of OP poisoning in the pediatric population, making it more challenging to diagnose correctly at the time of presentation. In a study, of the 20 patients, Zwiener and Ginsburg found that only four had an accurate diagnosis [6]. The differential diagnoses include bronchiolitis, bronchopneumonia, head trauma, opiate overdosage, and diabetic ketoacidosis. Bronchiolitis and bronchopneumonia are more common in the age group of our patient, and, because of the unavailability of exposure history, the clinician may not be able to make an accurate and prompt diagnosis [6].

The dosage, mode of exposure, and chemical potency all affect how quickly OP poisoning symptoms manifest. If the drug is particularly fat-soluble, it could take several days, but it could also take minutes in circumstances of significant ingestion or inhalation [9]. A decrease in serum and red blood cell cholinesterase activity is the hallmark diagnostic finding for OP poisoning. When its activity falls by 50% or more below standard laboratory values, OP poisoning is the likely diagnosis [10,11]. The serum cholinesterase activity of our patient was less than 20% of the control value, which supported and guided our patient's care.

The therapy is aimed at supporting ventilation as respiratory failure is the usual cause of death in these patients and more so in the pediatric age group. Atropine is a mainstay of treatment in OP poisoning, because of its anticholinergic properties. The recommended dosing is 0.02-0.05 mg/kg repeated every 5-10 min until symptom resolution; however, we refrained from repeating the dose at this frequency to avoid adverse effects because of extreme tachycardia in our patient. Similar actions have been taken in the literature on pediatric OP poisoning to prevent adverse effects [10]. However, this is to be tailor-made on a case-to-case basis, while keeping in mind that tachycardia alone is not a contraindication for the administration of atropine in such cases.

Pralidoxime (PAM) is a cholinesterase reactivator that aids in the reversal of respiratory muscle paralysis and muscle fasciculation by accelerating the restoration of enzyme activity at the neuromuscular junction. Within 24-48 hours of exposure, it should be given as an IV infusion over 20 minutes at a dose of 25-50 mg/kg [11]. If cholinergic symptoms return, the dose may be given again in one to two hours and thereafter at intervals of 10-12 hours [12]. We used the same dosage schedule for our patient.

## Conclusions

The presentation of OP poisoning in the pediatric age group is very varied with very few cases with typical presentations. Hence, it is a diagnostic challenge for emergency physicians. The more common differentials such as bronchitis and unavailability of a history of exposure add to the dilemma. In our patient too, only after asking repeatedly and directed questions, a history of exposure was identified. The life-threatening nature of the disease and the availability of antidotes for the treatment of OP poisoning presses on the need for early identification of the condition in all age groups, especially the pediatric age group. This case brings to light that leading questions and maintaining a high degree of suspicion is the key to early identification of the same. This further helps in directing the accurate and prompt management of these patients.

## References

[1] (2024). World Health Organization. Guidelines for establishing a poison centre. https://www.who.int/news/item/18-01-2021-who-guidelines-for-establishing-a-poison-centre.

[2] Karunarathne A, Bhalla A, Sethi A, Perera U, Eddleston M (2021). Importance of pesticides for lethal poisoning in India during 1999 to 2018: a systematic review. BMC Public Health.

[3] Naughton SX, Terry AV Jr (2018). Neurotoxicity in acute and repeated organophosphate exposure. Toxicology.

[4] Tafuri J, Roberts J (1987). Organophosphate poisoning. Ann Emerg Med.

[5] Gummin DD, Mowry JB, Beuhler MC (2021). 2020 annual report of the American Association of Poison Control Centers' National Poison Data System (NPDS): 38th annual report. Clin Toxicol (Phila).

[6] Zwiener RJ, Ginsburg CM (1988). Organophosphate and carbamate poisoning in infants and children. Pediatrics.

[7] Lifshitz M, Shahak E, Sofer S (1999). Carbamate and organophosphate poisoning in young children. Pediatr Emerg Care.

[8] Mortensen ML (1986). Management of acute childhood poisonings caused by selected insecticides and herbicides. Pediatr Clin North Am.

[9] Davies JE, Barquet A, Freed VH, Haque R, Morgade C, Sonneborn RE, Vaclavek C (1975). Human pesticide poisonings by a fat-soluble organophosphate insecticide. Arch Environ Health.

[10] Sharma N, Nin-Gonzalez R (2021). Organophosphate poisoning in a young child: a case report. Oxf Med Case Reports.

[11] Merrill DG, Mihm FG (1982). Prolonged toxicity of organophosphate poisoning. Crit Care Med.

[12] Bardin PG, Van Eeden SF (1990). Organophosphate poisoning: grading the severity and comparing treatment between atropine and glycopyrrolate. Crit Care Med.

